# The Japanese Catheter Ablation Registry (J‐AB): Annual report in 2019

**DOI:** 10.1002/joa3.12640

**Published:** 2021-10-07

**Authors:** Kengo Kusano, Teiichi Yamane, Koichi Inoue, Misa Takegami, Yoko M. Nakao, Michikazu Nakai, Koshiro Kanaoka, Koji Miyamoto, Yu‐ki Iwasaki, Seiji Takatsuki, Kohki Nakamura, Yoshihiro Miyamoto, Morio Shoda, Akihiko Nogami, Wataru Shimizu, J‐AB registry investigators

**Affiliations:** ^1^ Department of Cardiovascular Medicine National Cerebral and Cardiovascular Center Osaka Japan; ^2^ Division of Cardiology Department of Internal Medicine The Jikei University School of Medicine Tokyo Tokyo Japan; ^3^ Cardiovascular Division National Hospital Organization Osaka National Hospital Osaka Osaka Japan; ^4^ Department of Preventive Medicine and Epidemiology National Cerebral and Cardiovascular Center Suita Suita Japan; ^5^ Center for Cerebral and Cardiovascular Disease Information Open Innovation Center National Cerebral and Cardiovascular Center Suita Japan; ^6^ Department of Cardiovascular Medicine Nippon Medical School Tokyo Japan; ^7^ Department of Cardiology Keio University School of Medicine Tokyo Japan; ^8^ Department of Cardiology Gunma Prefectural Cardiovascular Center Tokyo Japan; ^9^ Clinical Research Division of Heart Rhythm Management Department of Cardiology Tokyo Women's Medical University Tokyo Japan; ^10^ Department of Cardiology Faculty of Medicine University of Tsukuba Tsukuba Japan

**Keywords:** catheter ablation, complication, J‐AB, REDCap, registry

## Abstract

The Japanese Catheter Ablation (J‐AB) registry, started in August 2017, is a voluntary, nationwide, multicenter, prospective, observational registry, performed by the Japanese Heart Rhythm Society (JHRS) in collaboration with the National Cerebral and Cardiovascular Center using a Research Electronic Data Capture system. The purpose of this registry is to collect the details of target arrhythmias, the ablation procedures, including the type of target arrhythmias, outcomes, and acute complications in the real‐world settings. During the year of 2019, we have collected a total of 80 795 procedures (mean age of 65.2 years and 66.4% male) from 425 participant hospitals. Detailed data are shown in Figures and Tables.

## Graphical abstract

J‐AB is a prospective nationwide multicenter registry designed to collect clinical variables, successful ablation rate and sort‐term outcome, aiming to register all catheter ablation cases performed in Japan. We successfully collected 80 795 procedures during the year of 2019.

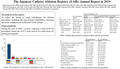

Catheter ablation has become an established therapy for the management of various cardiac arrhythmias and the procedure number has been dramatically increasing. However, little is known about the details of target arrhythmias, the ablation procedures, including the type of target arrhythmias, outcomes, and acute complications in the real‐world settings.

There are several preceding registries of catheter ablation, but the majority of which collected data from selected centers and/or selected arrhythmia and/or specified months to reveal the current status of ablations.^1^, ^2^, ^3^ Accordingly, we conducted a nationwide, multicenter, prospective, observational registry in Japan, named Japanese Catheter Ablation (J‐AB) registry, aiming to register all catheter ablation cases in Japan.^4^ This registry has been performed by the Japanese Heart Rhythm Society (JHRS) in collaboration with the National Cerebral and Cardiovascular Center using a Research Electronic Data Capture (REDCap) system. This study has been performed under the approval from the Institutional Review Board (IRB) of the National Cerebral and Cardiovascular Center (M28‐114‐7, approved at December 21, 2016), Japan, along with the IRBs of all participating hospitals. All participants were provided informed consent either by a written paper or by an opt out fashion and could withdraw their consent at any time. This study was also registered in the UMIN Clinical Trial Registry (UMIN 000028288) and ClinicalTrials.gov (NCT03729232). This J‐AB registry started in August 2017, since then the number of participating hospitals has increased to over 400 at the end of 2019. Annual data during the year of 2018 have been already reported,^5^ and now we report here the annual report of the results during the year of 2019. Figure 1 shows that the cumulative number of registered hospitals and the patients during the year of 2019. Figure 2 shows that the number and rate of the target arrhythmias. AF procedure was the most common (73.8% of all ablation procedures) in 2019. Patient characteristics, acute outcomes, and acute complications of all and AF procedures are shown in Tables 1, 2, and 3, respectively. Compared to the previous reports in Japan and other countries,^1^, ^2^, ^3^ acute complications during hospitalization were similar or low. In the Spanish Catheter Ablation Registry, the rate of all complications was 2.0%‐2.6% for all ablation procedures, and 3.4%‐5.1% for AF ablation. In the US report, the overall complication rate was 5.46% and the in‐hospital mortality rate was 0.15% for AF ablation. In the J‐CARAF during the years from 2011 to 2016, total major complications occurred in 3.0% of the AF ablation procedures.

**FIGURE 1:**
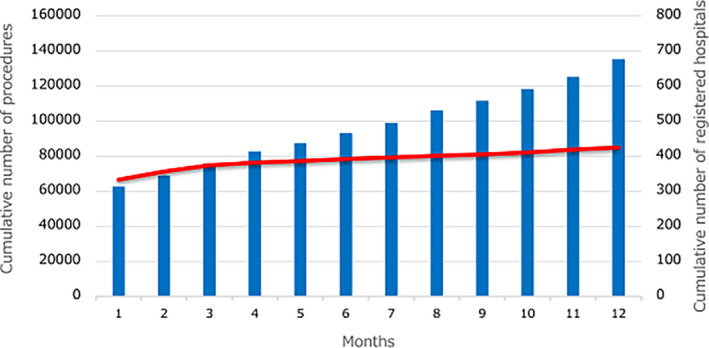
Cumulative number of registered hospitals (red line) and the patients (blue bars) during the year of 2019

**FIGURE 2:**
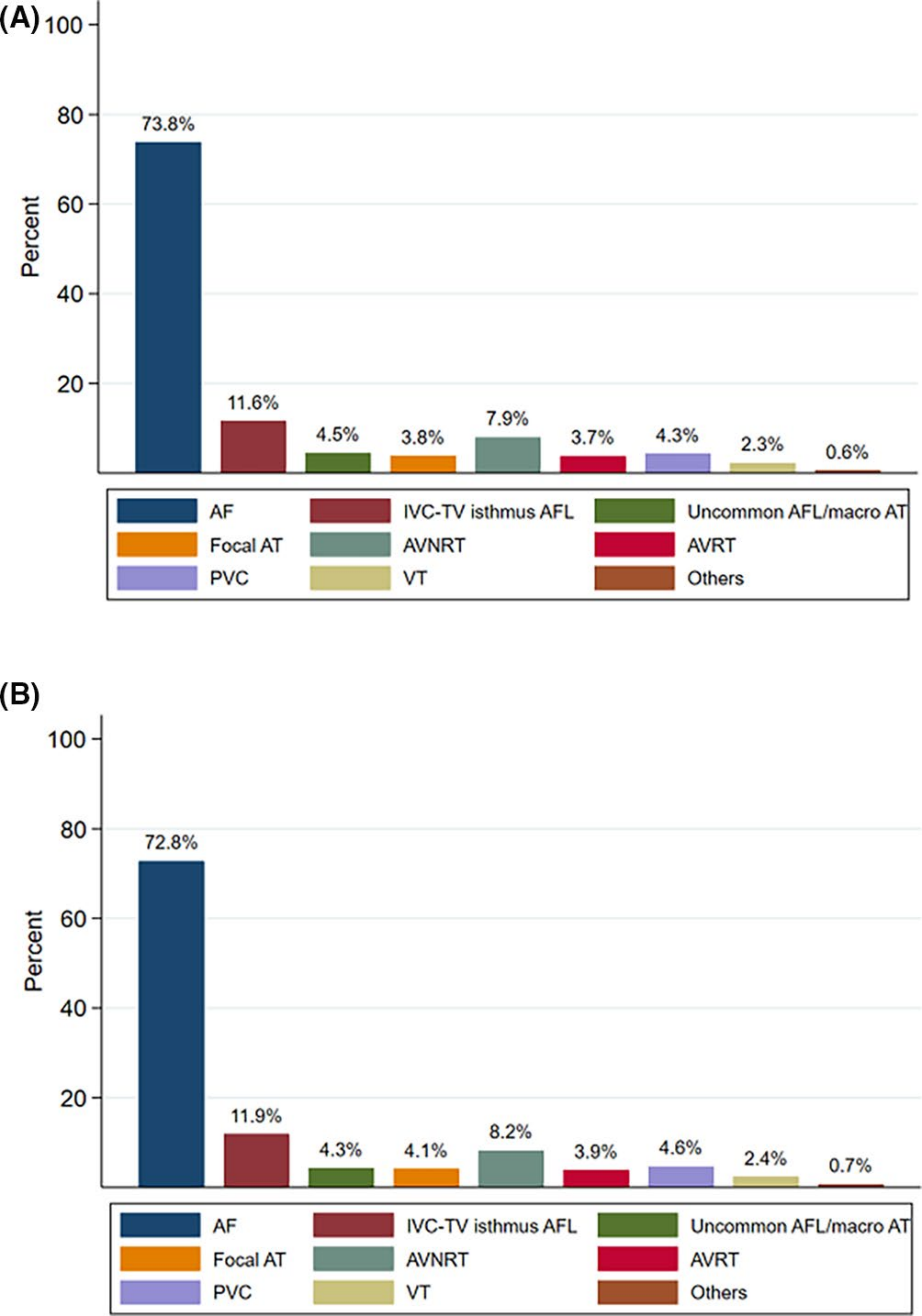
The proportion of the target arrhythmias in the J‐AB registry. (A) The J‐AB registry 2019 (80,795 procedures). (B) The J‐AB registry 2018 (55,525 procedures). Abbreviations: AF, atrial fibrillation; IVC, inferior vena cava; TV, tricuspid valve; AFL, atrial flutter; AT, atrial tachycardia; AVNRT, atrioventricular nodal reentrant tachycardia; AVRT, atrioventricular reentrant tachycardia; PVC, premature ventricular contraction; VT, ventricular tachycardia

**TABLE 1:** Patient characteristics

		Atrial fibrillation (AF)			Atrial flutter (AFL)/Atrial tachycardia (AT)			
	All procedures	All AF	Paroxysmal AF (PAF)	Non‐PAF	All AFL/AT	IVC‐TV isthmus‐dependent AFL^a^	Uncommon AFL macro‐AT^a^	Focal AT^a^
N	80 795	59 624	35 343	24 138	13 661	8838	3132	2686
Age, mean ± SD	65.2 ± 13.1	66.9 ± 10.5	67.1 ± 10.9	66.6 ± 10.1	67.6 ± 12.9	68.1 ± 12.0	68.9 ± 11.9	64.9 ± 15.9
Gender, male	53 657 (66.4%)	41 558 (69.7%)	23 220 (65.7%)	18 244 (75.6%)	9213 (67.4%)	6689 (75.7%)	1819 (58.1%)	1250 (46.5%)
BMI, mean ± SD	23.9 ± 3.8	24.2 ± 3.7	23.9 ± 3.6	24.7 ± 3.8	23.5 ± 3.8	23.6 ± 3.7	23.5 ± 3.8	22.7 ± 3.8
Heart diseases	15 505 (19.2%)	10 816 (18.1%)	5649 (16.0%)	5153 (21.4%)	4033 (29.5%)	2520 (28.5%)	1385 (44.2%)	629 (23.4%)
IHD	5110 (6.3%)	3609 (6.1%)	2167 (6.1%)	1437 (6.0%)	1100 (8.1%)	784 (8.9%)	267 (8.5%)	135 (5.0%)
Cardiomyopathy	4592 (5.7%)	3249 (5.4%)	1296 (3.7%)	1951 (8.1%)	1002 (7.3%)	609 (6.9%)	334 (10.7%)	158 (5.9%)
Valve disease	2538 (3.1%)	1501 (2.5%)	731 (2.1%)	763 (3.2%)	1229 (9.0%)	691 (7.8%)	560 (17.9%)	187 (7.0%)
CHD	985 (1.2%)	430 (0.7%)	242 (0.7%)	188 (0.8%)	527 (3.9%)	306 (3.5%)	231 (7.4%)	103 (3.8%)

				Ventricular tachycardia (VT)		
	Atrioventricular nodal reentrant tachycardia	Atrioventricular reentrant tachycardia	Premature ventricular contraction	VT due to ischemic cardiomyopathy	VT due to nonischemic cardiomyopathy	VT due to CHD
N	6409	3000	3501	433	502	18
Age, mean ± SD	58.7 ± 16.7	48.9 ± 20.0	57.8 ± 16.1	68.6 ± 10.1	63.6 ± 13.1	41.7 ± 13.7
Gender, male	2701 (42.1%)	1926 (64.2%)	1894 (54.1%)	404 (93.3%)	396 (78.9%)	16 (88.9%)
BMI, mean ± SD	22.9 ± 3.9	23.0 ± 3.9	23.7 ± 3.9	24.2 ± 3.8	23.5 ± 4.0	25.6 ± 5.1
Heart diseases	479 (7.5%)	195 (6.5%)	633 (18.1%)	396 (91.5%)	452 (90.0%)	17 (94.4%)
IHD	155 (2.4%)	55 (1.8%)	221 (6.3%)		16 (3.2%)	0 (0%)
Cardiomyopathy	71 (1.1%)	33 (1.1%)	252 (7.2%)	14 (3.2%)		0( 0%)
Valve disease	63 (1.0%)	24 (0.8%)	60 (1.7%)	18 (4.2%)	22 (4.4%)	0( 0%)
CHD	45 (0.7%)	29 (1.0%)	23 (0.7%)	1 (0.2%)	6 (1.2%)	

Abbreviations: BMI, Body Mass Index; CHD, congenital heart disease; IHD, ischemic heart disease; SD, Standard Deviation

^a^ Multiple choices allowed.

**TABLE 2:** Acute outcomes

Pulmonary vein isolation for atrial fibrillation (n = 58 429)	
Ablation system n (%)	
RF alone	43 047 (73.67%)
Balloon alone (Cryo, hot, laser)	10 464 (17.91%)
RF + Balloon combination	4586 (7.85%)
Others	168 (0.29%)
Missing	164 (0.28%)
Patient with a first session (n = 47 726)	
Success	47 462 (99.45%)
Unsuccess	186 (0.39%)
Already isolated	60 (0.13%)
Unknown	18 (0.04%)
Patient with second session (n = 8863)	
Success	7448 (84.03%)
Unsuccess	19 (0.21%)
Already isolated	1388 (15.66%)
Unknown	8 (0.09%)
Additional ablation only	577 (6.09%)
Patient with third session (n = 2090)	
Success	1138 (64.40%)
Unsuccess	4 (0.23%)
Already isolated	625 (35.37%)
Additional ablation only	319 (15.26%)
IV‐TV isthmus‐dependent atrial flutter (n = 8838)	
Success	8776 (99.30%)
Unsuccess	59 (0.67%)
Unknown	3 (0.03%)
Uncommon atrial flutter/atrial tachycardia (n = 3132)	
Complete success	2650 (84.61%)
Partial success	319 (10.19%)
Unsuccess	103 (3.29%)
Unknown	60 (1.92%)
Focal atrial tachycardia (n = 2686)	
Complete success	2238 (83.32%)
Partial success	313 (11.65%)
Unsuccess	101 (3.76%)
Unknown	34 (1.27%)
Atrioventricular nodal reentrant tachycardia by slow‐fast (n = 5574)	
Complete success	5457 (97.90%)
Partial success	70 (1.26%)
Unsuccess	29 (0.52%)
Unknown	18 (0.32%)
Atrioventricular nodal reentrant tachycardia by fast‐slow (n = 581)	
Complete success	558 (96.04%)
Partial success	18 (3.10%)
Unsuccess	3 (0.52%)
Unknown	2 (0.34%)
Atrioventricular nodal reentrant tachycardia by other (n = 581)	
Complete success	339 (90.40%)
Partial success	20 (5.33%)
Unsuccess	7 (1.87%)
Unknown	9 (2.40%)
Atrioventricular reentrant tachycardia by kent (n = 2951)	
Complete success	2840 (96.24%)
Unsuccess	85 (2.88%)
Unknown	26 (0.88%)
Premature ventricular contraction (n = 3501)	
Complete success	2642 (75.46%)
Partial success	602 (17.20%)
Unsuccess	228 (6.51%)
Unknown	29 (0.83%)
Idiopathic ventricular tachycardia (n = 781)	
Complete success	595 (76.18%)
Partial success	122 (15.62%)
Unsuccess	42 (5.38%)
Unknown	22 (2.82%)
Ventricular tachycardia due to ischemic cardiomyopathy (n = 433)	
Complete success	272 (62.82%)
Partial success	117 (27.02%)
Unsuccess	20 (4.62%)
Unknown	24 (5.54%)
Ventricular tachycardia due to nonischemic cardiomyopathy (n = 502)	
Complete success	289 (57.57%)
Partial success	156 (31.08%)
Unsuccess	40 (7.97%)
Unknown	17 (3.39%)
Ventricular tachycardia due to CHD (n = 18)	
Complete success	10 (55.56%)
Partial success	7 (38.89%)
Unsuccess	1 (5.56%)

Abbreviations: CHD, congenital heart disease; IVC, inferior vena cava; RF, radiofrequency ablation; TV, tricuspid valve.

**TABLE 3:** Acute complications: All procedures and AF procedures

	All procedures	AF procedures
N	80 795	59 624
Complications during hospitalization	2023 (2.50%)	1633 (2.74%)
Major bleeding (BARC>=2)	902 (1.12%)	700 (1.17%)
Cardiac tamponade	532 (0.66%)	380 (0.64%)
Embolism	149 (0.18%)	128 (0.21%)
Phrenic nerve paralysis	212 (0.26%)	205 (0.34%)
Esophagus	147 (0.18%)	146 (0.24%)
Esophagus ulcer	20 (0.02%)	19 (0.03%)
Gastric hypomotility	127 (0.16%)	127 (0.21%)
Atrioesophageal fistula	0 (0)	0 (0)
Pericarditis	99 (0.12%)	84 (0.14%)
Sick sinus syndrome	134 (0.17%)	110 (0.18%)
Atrioventricular block	65 (0.08%)	17 (0.03%)
Death during hospitalization	89 (0.11%)	34 (0.06%)
Cardiac death	58 (0.07%)	18(0.03%)
Related to ablation therapy	2 (0.002%)	1 (0.002%)
Non‐cardiac death	31 (0.04%)	16(0.03%)
Related to ablation therapy	1 (0.001%)	0 (0)

Abbreviations: AF, atrial fibrillation; BARC, Bleeding Academic Research Consortium.

## CONFLICT OF INTEREST

Kengo Kusano: Speaker honoraria from DAIICHI SANKYO COMPANY, Ltd., Japan, Bristol‐Myers Squibb, Biotronik Japan, and Medtronic Japan, and research grants from Medtronic Japan and EP‐CRSU Co., Ltd. Teiichi Yamane: Speaker honoraria from DAIICHI SANKYO COMPANY, Ltd., Japan, Boerringer Ingelheim, Abbott Japan, Medtronic Japan, and Kaneka Corporation and research grants from Boehringer Ingelheim. Koichi Inoue: Speaker honoraria from DAIICHI SANKYO COMPANY, Ltd., Japan, Bristol‐Myers Squibb, Bayer Yakuhin, Nihon Boehringer Ingelheim, Johnson and Johnson KK, and Medtronic Japan. Koji Miyamoto: Research grant from Japan LifeLine, Abbott Japan, Speaker honoraria from Daiichi Sankyo, Boerringer Ingelheim, Bayer, Bristol‐Myers Squibb, Pfizer, Abbott Japan, and Medtronic Japan. Yu‐ki Iwasaki: Research grant from Daiichi Sankyo, Seiji Takatsuki: Research grant from Japan Lifeline, honoraria from Medtronic Japan, Daiichi Sankyo. Morio Shoda: Speaker honorarium from Medtronic Japan, and financial endowments to our clinical research division from Biotronik Japan, Medtronic Japan, Boston Scientific Japan, and Abbott Japan. Akihiko Nogami: Speaker honoraria from Abbott, Biosense Webster, and Daiichi‐Sankyo; an endowment from Medtronic and DVX. Wataru Shimizu: Research grant from Daiichi Sankyo Co, Ltd., and Nihon Boehringer Ingelheim, and Speaker honoraria from Daiichi Sankyo Co, Ltd., Bristol‐Myers Squibb Co, Ltd, Bayer Yakuhin Co, Ltd, Nihon Boehringer Ingelheim, Ono Pharmaceutical Co, Ltd, Otsuka Pharmaceutical Co, Ltd, Novartis Pharma KK, and Medtronic Japan. None: MT, YMN, M.K, M.N, K.K, K.N, and YM
